# Langmuir Probe Perturbations during In Situ Monitoring of Pulsed Laser Deposition Plasmas

**DOI:** 10.3390/ma15082769

**Published:** 2022-04-09

**Authors:** Ștefan-Andrei Irimiciuc, Sergii Chertopalov, Michal Novotný, Valentin Craciun, Jan Lancok, Maricel Agop

**Affiliations:** 1 National Institute for Laser, Plasma and Radiation Physics–NILPRP, 409 Atomistilor Street, 077125 Bucharest, Romania; stefan.irimiciuc@inflpr.ro (Ș.-A.I.); valentin.craciun@inflpr.ro (V.C.); 2 Institute of Physics of the Czech Academy of Sciences, Na Slovance 1999/2, 18221 Prague, Czech Republic; chertopalov@fzu.cz (S.C.); novotnym@fzu.cz (M.N.); 3 Extreme Light Infrastructure for Nuclear Physics, IFIN-HH, 077125 Magurele, Romania; 4 Department of Physics, “Gh. Asachi” Technical University of Iasi, 700050 Iasi, Romania; magop@tuiasi.ro; 5 Romanian Scientists Academy, 54 Splaiul Independentei, 050094 Bucharest, Romania

**Keywords:** laser-produced plasmas, Langmuir probe, perturbative regime, multifractal model, strange attractors

## Abstract

The recent advancements in pulsed laser deposition (PLD) control via plasma diagnostics techniques have been positive and raised questions on the limitation of some techniques, such as the Langmuir probe (LP). The particularities of laser-produced plasma can lead to incorrect interpretation of collected electrical signal. In this paper, we explored the limitations of LP as a technique for in situ PLD control by performing investigations on several metallic plasmas, expanding in various Ar atmosphere conditions. Sub-microsecond modulation was seen in the reconstructed *IV* characteristics attributed to non-equilibrium dynamics of the ejected charges. A perturbative regime was recorded for Ar pressures higher than 2 Pa, where ionic bursts were observed in the electron saturation region. This perturbation was identified as a plasma fireball. A non-linear multifractal model was developed here to explore these new regimes of the LP. The strange attractors characterizing each fireball were reconstructed, and their evolution with the Ar pressure is discussed. Both short- and long-time non-linear behavior were correlated via probe bias, and the pressure effect on the strange attractor’s defining the fireball-like behavior was investigated. A good correlation was noticed between the simulated data and experimental findings.

## 1. Introduction

Some of the first electrical measurements on laser-produced plasmas date back to the early 1960s, a short time after the first implementation of a ruby laser as source to generate transient plasmas [1,2]. There were two approaches reported in the early days of laser-produced plasma diagnostics: collecting the electrical current from the generated plasma or irradiated target. The Langmuir probe technique has become, in the past 15 years, a staple of laser-produced plasmas (LPP) diagnostics, based on the groundbreaking work of Wood et al. [3,4], Luney et al. [5,6], and Schou et al. [7]. The approach was first built around understanding the temporal traces of the current. There, a series of models were used to interpret the dynamic of the ejected plumes [8,9,10,11]. Recently, based on new information mainly related to the dissipative behavior of LPP, a scattering [12] model and one based on a multifractal theory of motion were proposed [13]. The presence of multiple maxima in the ionic current of the probe, or of even more complex features, explained [14,15,16] as ionic oscillations, have challenged the accepted view of ion dynamics in LPP, especially in high fluence irradiation or complex stoichiometric targets. This was explained by considering different ablation mechanisms, complex acceleration, and scattering dynamics during the expansion of the LPP. To confirm this new paradigm, alternative techniques have been implemented, either aimed towards complementarity, such as space- and time-resolved optical emission spectroscopy [17,18] and intensified coupled charged device (ICCD) fast camera imagining [19,20,21], or towards increasing the knowledge offered by LP or other electrostatic sensors by mass spectrometry [22,23]. Alternatively, the sweeping technique was implemented for a time-resolved analysis of specific plasma parameters, as defined in the physics of plasma discharges [24,25]. Considering the particularities of LPP, certain limitations will arise: geometrical limitations induced by the ratio between the probe electrode and dimension of the plasma, as the probe needs to be considerably smaller than the investigated area/volume; the measuring distance needs to be large enough, so that the plasma volume is larger than the dimension of the probe; and limitation on the applied biases, as higher values and favorable plasma density conditions can induce sputtering of the probe and contamination of the plasma. These limitations are always mentioned, but rarely truly explored in literature, since avoiding them easily validates that the probe measurement can be further used to gain information on the fundamentals of laser ablation, as well as about the pulsed laser deposition technique. Understanding the implementation limits for a relatively simple technique, such as LP, can become of outmost importance for real-time in situ monitoring of the deposition process, as well as the steps needed to be undertaken to transfer PLD towards industry. A brief report was performed by our group in [26], where we discussed the possibility of a fireball-like structure formation on the probe in high Ar pressure conditions, when depositing nanostructured Ag films. 

In this paper, we present a detailed study of LP perturbation during ion current measurements in laser-produced plasmas, over a wide range of metals and Ar pressures in identical irradiation conditions. A time-resolved LP technique is implemented to explore the short-time dynamics of laser-produced plasma, while pressure-dependent measurements were aimed towards understanding the limits of LP implementation in real PLD conditions. A theoretical model is developed in the framework of a multifractal theory of motion, in order to explore the nature of the perturbations. 

## 2. Results

**Figure 1:**
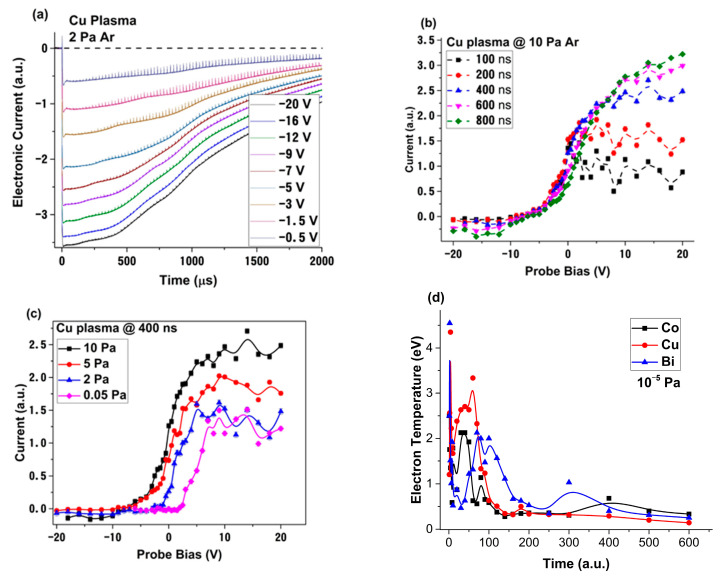
Selected signals from Cu plasma in 2 Pa of Ar (**a**), the IV characteristic of Cu plasma at various 10 Pa of Ar (**b**) and various moments in time (**c**), as well as the electron temporal evolution for Co, Cu and Bi plasmas (**d**) (lines are guides for the eyes).

When investigating the temporal traces recorded by the LP method, there are a plethora of reports [27,28,29,30,31,32,33] where the LP technique is discussed and implemented. For all the investigated plasmas presented in this work, with the increase of the Ar background pressure, the overall electronic charge collected increased exponentially, with an average factor of 10 for all metals and the mention that Bi plasma has permanently higher electron density, when compared to the other plasmas. A closer look into the shape of the signals at higher pressures (above 0.5 Pa, Figure 1a) leads to the observation of a periodic pulsed behavior. Based on the shape of the oscillation and probe biases, for which they are measured, each peak would correspond to a localized ionic emission in the plasma volume surrounding the probe. The frequencies of these oscillations are in the kHz regime, consistent with ionic oscillation in low temperature plasma. The characteristics of the observed oscillations correspond to a fireball-like behavior, as it was described by Dimitriu et al. [34] and Schrittwieser et al. [35]. According to them, a fireball is formed on the surface of an electrode immersed in a low temperature plasma, for a wide range of electron densities and working gases [36]. The generated fireball contains an ionic core, shielded from the LPP by an unstable double layer. When the double layer breaks down, a surge of ions will be ejected in the plasma, which is immediately followed by the reformation of the double layer. According to previous work performed on fireball dynamics [37,38], the ionic core contains only working gas species, and there is no reported sputtering of the electrode. This is in line with our report from [26], where the increase of the working gas during LP monitored PLD of Ag did not generate any impurities in the deposited films. 

By collecting the ionic and electronic current characteristics, in a wide range of probe biases (±20 V in 50 points), and implementing the time-sweeping method [24,25], we were able to reconstruct the IV characteristics (Figure 1b,c). The laser generated plasmas were investigated for a wide range of temporal points (up to 600 μs). Examples of the temperature evolution in time are given in Figure 1d, where we see an exponential decrease for sub μs sequences, followed by secondary maxima in the μs and tens of μs region. In the <1 μs region, the reconstructed IV characteristics present a strongly modulated electronic saturation region, starting from ~1 V. Similar behavior was reported in [14], where the authors plotted the overall collected charge as a function of voltage. The data reported here proves that the modulation of the electronic current characterizes, in fact, particular time sequences. In Figure 1b,c, the transition from modulated to classical shapes of IV characteristics for a Cu plasma is presented, with the mention that this is a characteristic of all investigated metallic plasmas. With the LPP expansion, it is observed that the modulated part vanishes at about 1 μs, presumably when a dynamic equilibrium is reached. The temporal framing of the process could indicate that, during expansion in the first hundreds of ns, the plasma has a dynamic dominated by strong nonlinear behaviors. These modulated dynamics of the electron saturation charge are strongly influenced by the background pressure and become a dominant feature for Ar pressure above 0.5 Pa, when the dynamics defining the LPP evolution shift from free expansion to collisional regimes. This threshold represents a clear limit, for which the LP measurements start to be dominated by nonlinear effects. Moreover, the addition of Ar gas shifts the floating potential of the plasmas towards negative values, from 2 V to −6 V for Cu, from 0.5 to −4 for Bi, from 3 to −9V for Co, and from 1.5 to −5 V for Ag. 

**Figure 2:**
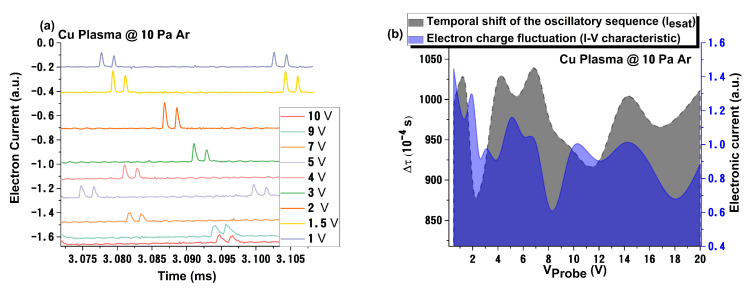
Oscillatory signal variation with the applied (**a**) and probe bias variations of the oscillation shift and overall electronic charge (**b**).

To explore the fireball-like phenomena induced during LP measurements, we have represented the electron current collected, versus time, for various biases (Figure 2). We observe that the positive peaks in the current are not synchronized and appear at different random moments of time for specific probe biases. This dependence is represented in Figure 2b (grey). The modulation in electronic charge, as function of probe bias, well-overlaps the temporal shift of the oscillatory sequence. This means that the two non-linear phenomena are correlated and have a similar nature. It was also found that the increase of working pressure enhances the correlation between the two non-linear processes. This means that the Ar gas acts like a membrane that can build coherent correlations between phenomena that manifest themselves at different temporal scales (<1 μs for the *I_esat_* and *ms* scales for fireball-like oscillations).

**Figure 3:**
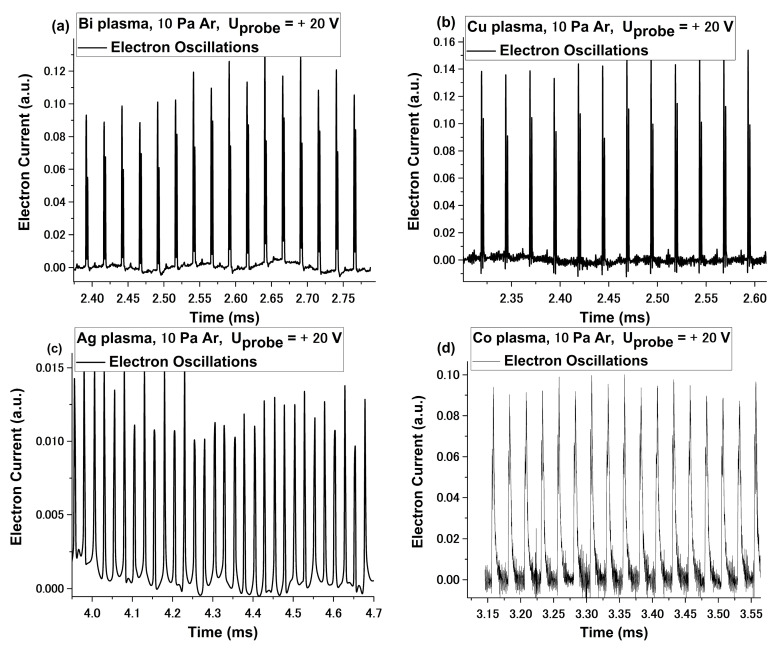
Example of oscillatory regime for Bi (**a**), Cu (**b**), Ag, (**c**) and Co (**d**) plasmas at (+20 V) at 10 Pa of Ar.

In Figure 3, we have represented the oscillatory sequence subtracted from the electronic temporal traces for all the investigated plasmas at 10 Pa of Ar and +20 V applied bias. The same behavior is, thus, seen for Bi, Co, Cu, and Ag in high pressure conditions (above 0.5Pa Ar). For the investigated plasmas, local fluctuation of the background noise, overlapped on the clear oscillatory signal, can be seen. The variation of the background is not correlated with any changes during the measurement, in terms of probe bias, background gas pressure, or target nature. The values of the oscillation frequencies determined here (in the kHz regime) are well in line with ionic-type oscillations and previous reports of oscillatory fireball-like structures [37]. The amplitude of these oscillations increases with the addition of Ar atmosphere. The dependences on the background gas and probe bias, presented in Figure 1, Figure 2 and Figure 3 and interpretation given in [38,39] on the properties of plasma fireballs, lead to the conclusion that the fireball-like structure that forms around the probe is of Ar gas nature, as initially reported by us in [26]. However, there is also a strong dependence on the nature of the irradiated target. In Figure 3, we observed a difference in the shape and frequency of the oscillation. For the Co plasma, we observed that the oscillatory signal was asymmetric and of the relaxation type, while the ones for Cu were symmetric and of the pulse type. These significant differences underline an important aspect of these fluctuations: although clearly induced by the pressure increase of Ar atmosphere, they are of metallic nature. This means that, when the fireball-like structure forms on the probe, the trapped ions inside the structure are metallic, rather than Ar ions, as was initially assessed. The true nature of the laser-produced plasmas fireball core is rather difficult to elucidate, due to its transient nature and the strong density gradients and spatial distribution of the ablated and background gas particles.

**Figure 4:**
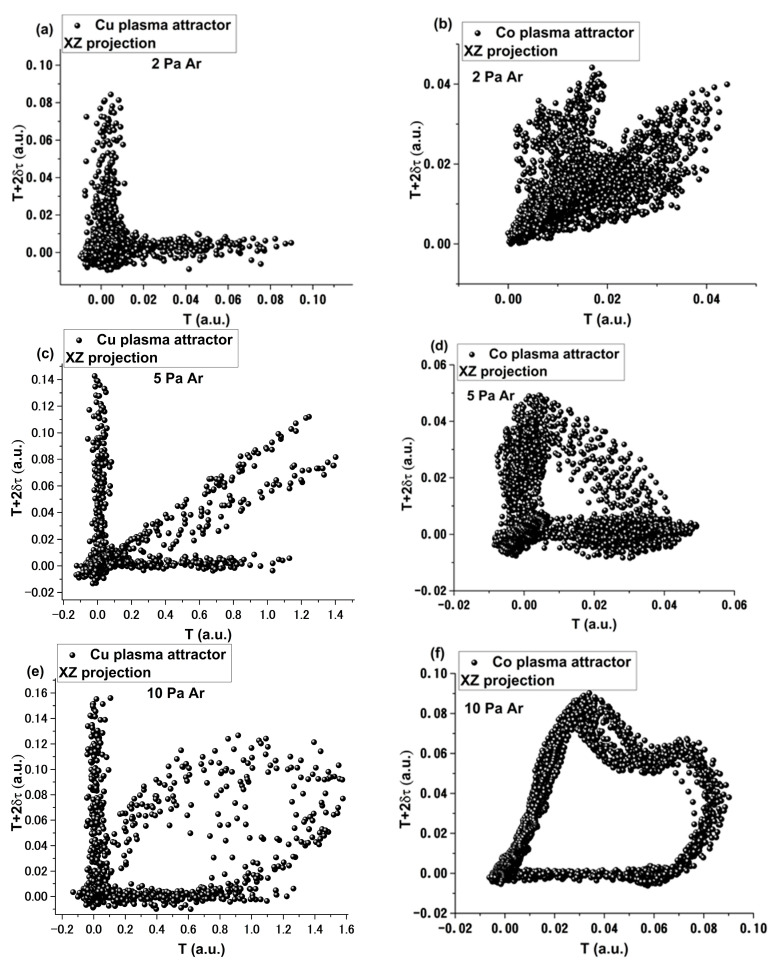
The 2D representation of the attractor associated to the oscillatory behavior observed for Cu at 2 Pa (**a**), 5 Pa (**c**) and 10 Pa (**e**) and Co at 2 Pa (**b**), 5 Pa (**d**) and 10 Pa (**f**).

Further insight into the nonlinear properties of these oscillations and their stability, in terms of chaoticity and fractality of the system, can be extracted from the evolution of the strange attractors attributed to each oscillatory regime. Using the standard approach from [40], which implies the use of the autocorrelation function and Theiler temporal window, the strange projection of the strange attractors in the *T* and *T* + 2*δτ*, where the *T* is the time of the system (fireball-like structure), and *δτ* is the time period defined by the first zero crossing of the autocorrelation function. The selected results are presented in Figure 4 for Co and Cu, with the mention that the similar analysis was performed for Ag and Bi cases. In Figure 4, we observed that the attractor of the fireball-like structure exhibited two *arms*, defining two distinct trajectories in this correlation phase. This was seen for all investigated plasmas. With the increase of Ar pressure, we observed that, for Co plasma, a bridge formed between the two arms, with a clear correlation between them. This means that the two trajectories are now connected and can influence one another. For the Cu and Ag cases (Ag not shown here), a third trajectory was defined in between the original arms. With the increase of Ar pressure, up to 10 Pa, the central trajectory extends and becomes the bridge between the arms of the attractor. The transition observed here is clearly correlated with the thermal and kinetic energy of the metallic LPP.

**Figure 5:**
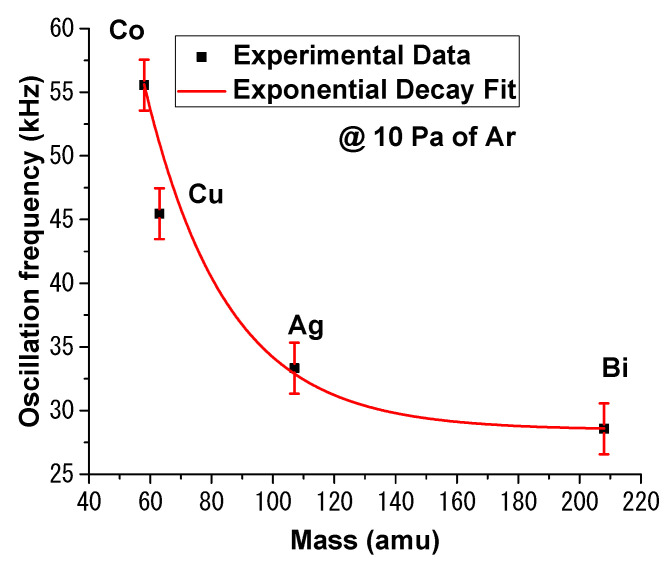
Oscillation frequency dependence on the mass plasma species.

Finally, to confirm the nature of the perturbation, we have represented the oscillation frequency as a function of the target atomic mass. The results are presented in Figure 5. The experimental data was fitted with an exponential decay-type function (R = 97.9%). This result confirmed that the perturbation was of metallic nature and proportional to the atomic mass. The exponential dependence on the atomic mass means these were plasma-type oscillations. These results contradict the report from [14], where an oscillatory regime was discussed over a wide range of plasmas; an exponential increase proportional with the atomic mass was seen, and oscillations were in the MHz regime, with no interpretation of the surprising evolution. 

### 2.1. Theoretical Model for LP Perturbator Regimes 

The theoretical approach developed in this paper will substitute the complexity of the system (laser-produced plasma), with various concepts of fractality. The fractality, in the framework of transient plasmas generated by laser ablation, is defined by considering, at a microscopic domain, that between two consecutive collisions the trajectory of a structural unit (i.e., electrons, ions, atoms, molecule, clusters, etc.) is a straight line. In the initial stages of ablation, the plasma density is extremely high and the volume is small, the incipient factors in the ablation process are defined by a higher number of collisions, and the trajectories of the laser ablation plasma structural units are “broken”. Projecting this scenario in the multifractal model, we will assume that the system has an uncountable set of points in the space–time coordinates that define fractured lines, whose non-linearity factors depend on the fractal dimension. In reality, the transient plasmas are inhomogeneous and anisotropic media with strong density and energy gradients; therefore, various plasma volumes will be described by different fractal dimensions simultaneously, which means that the ejected particles can be best defined by multifractal curves, with associated multifractal dimensions. This means that, in the framework of our model, we can assume the movement of structural units on continuous, but non differentiable, multifractal curves. This implies that the single variable dependencies are replaced, in certain approximations, with functions (i.e., the averaging over various scale resolutions). Therefore, the mathematical variable, used here to characterize LPP dynamics, can act as a limit of a specific class of functions, and said variable will be non-differentiable for null-scale resolutions and differentiable in any other possible cases [41,42]. 

Due to the large temporal scales, for which relevant dynamics in laser ablation process can be defined (fs-ms) [43], the deterministic trajectories of any structural units of the plasma are substituted by a class of virtual trajectories. In this development, the concept of definite trajectories is replaced by the one of probability density. Thus, by taking into account all the particularities of the transient plasma dynamics projected in the multifractal environment, plasma stochasticity will define the fractality of the mathematical object (which is assimilate to the LPP) and become operational through the scale relativity theory of motion (SRT) [42,43]. 

In the following, it is considered that only the plasma structural units fractalization cases via Markov-type stochastic processes [41,44]. Thus, in the framework of SRT, a Schrodinger-type description, at various scale resolutions (multifractal Schrodinger’s descriptions), as well as a Madelung-type description in the same range of scale resolutions (multifractal Madelung’s description), can be simultaneously implemented. Both approaches are complementary in the description of transient plasma dynamics. Let us consider the multifractal Schrodinger equation for the 1-dimensional case, written as:

(1) $$\mu^{2}\partial_{x}\partial^{x}\Psi \left(x,t\right)+i\mu \partial_{t}\Psi \left(x,t\right)=0$$

where

(2) $$\partial_{x}\partial^{x}=\frac{\partial }{\partial x}\left(\frac{\partial }{\partial x}\right),\partial_{t}=\frac{\partial }{\partial t},\mu =\lambda {\left(dt\right)}^{\left[\frac{2}{f\left(\alpha \right)}\right]-1},\;i=\sqrt{-1}$$

In Equations (1) and (2), $x^{l}$ is the multifractal spatial coordinate, *t* is the non-multifractal time coordinate (with the role of affine parameters of the motion curves), *dt* is the scale resolution, *λ* is the differentiable-nondifferentiable transition associated constant, f(α) is the singularity spectrum of α order, with α the singularity index, and it represents the functional of fractal dimension *D_F_*, $\alpha =\alpha \left(D_{F}\right)$, and Ψ is the multifractal state function. By using the singularity spectrum, it is possible to reach a wide reach of interactions classes in laser ablation plasma dynamics. 

The solution of the one-dimensional multifractal Schrödinger Equation (1) can be written in the form:

(3) $$\Psi \left(x,t\right)=\frac{1}{\sqrt{t}}\exp\left(i\frac{x^{2}}{4\mu t}\right)$$

and is defined, of course, up to an arbitrary multiplicative constant. 

As such, the general solution of Equation (1) can be written as a linear superposition, in the form:

(4) $$\Psi \left(x,t\right)=\frac{1}{\sqrt{t}}\overset{+\infty }{\underset{-\infty }{\int }}u\left(y\right)\exp\left[i\frac{{\left(x-y\right)}^{2}}{4\mu t}\right]dy$$

Now, if u(y) is an Airy function of multifractal-type, then Ψ(x,t) retains this property, in the sense that its amplitude is an Airy multifractal function. Indeed, in this case, there will be:

(5) $$u\left(y\right)\equiv Ai\left(y\right)=\frac{1}{2\pi }\overset{+\infty }{\underset{-\infty }{\int }}\exp\left[i\left(\frac{\omega^{3}}{3}+\omega y\right)\right]d\omega$$

in such a way as the state Function (4) will be written in the form:

(6) $$\Psi \left(x,t\right)=\frac{1}{2\pi \sqrt{t_{u,s}}}\overset{+\infty }{\underset{-\infty }{\int }}\exp\left\{i\left[\frac{\omega^{3}}{3}+\omega y+\frac{{\left(x-y\right)}^{2}}{4\mu t}\right]\right\}dyd\omega$$

If, at first, the integration will be carried out after y, up to a multiplicative constant, the results are:

(7) $$\Psi \left(x,t\right)=\frac{1}{2\pi }\overset{+\infty }{\underset{-\infty }{\int }}\exp\left[i\left(\frac{\omega^{3}}{3}+\omega x-\mu t\omega^{2}\right)\right]d\omega$$

The final result is obtained based on a special relation developed in [45], and it is:

(8) $$\Psi \left(x,t\right)=\left[Ai\left(kx-v^{2}t^{2}\right)\right]\exp\left[i\nu t\left(kx-\frac{2}{3}v^{2}t^{2}\right)\right]$$

With

(9) $$v=k^{2}\mu$$

In these conditions, if Ψ is chosen in the form:

(10) Ψ(x,t)=A(x,t)exp[iϕ(x,t)]

where A(x,t) is an amplitude, and Φ(x,t) is a phase. By identifying the amplitude and phase in (8), there will be:

(11) $$A\left(x,t\right)=Ai\left(kx-v^{2}t^{2}\right),\;\;\phi \left(x,t\right)=vt\left(kx-\frac{2}{3}v^{2}t^{2}\right)$$

Taking into account the asymptotic behavior of the function Ai(z), in its general form:

(12) $$Ai\left(z\right)\sim \{\begin{array}{ll} \frac{1}{2\pi^{1/2}}z^{-1/4}\exp\left(-\frac{2}{3}z^{3/2}\right), & z\to +\infty \\ \frac{1}{\pi^{1/2}}{\left|z\right|}^{-1/4}\sin\left(\frac{2}{3}{\left|z\right|}^{3/2}+\frac{\pi }{4}\right), & z\to -\infty \end{array}$$

the state function (10), with (11) function, in the asymptotic limit $\Psi \to \Psi_{A}$, becomes:

(13) $$\Psi_{A}\sim \{\begin{array}{c} \frac{1}{2\pi^{1/2}}{\left(kx-v^{2}t^{2}\right)}^{-1/4}\exp\left[-\frac{2}{3}{\left(kx-v^{2}t^{2}\right)}^{3/2}+ivt\left(kx-\frac{2}{3}v^{2}t^{2}\right)\right]\\ \frac{1}{\pi^{1/2}}{\left|kx-v^{2}t^{2}\right|}^{-1/4}\sin\left[\frac{2}{3}{\left|kx-v^{2}t^{2}\right|}^{\frac{3}{2}}+\frac{\pi }{4}\right]\exp\left[ivt\left(kx-\frac{2}{3}v^{2}t^{2}\right)\right] \end{array}$$

In the following, let us address the correspondence with the multifractal Madelung representation of the laser ablation plasma dynamics. By substituting (10) in (1), by means of direct calculation, the following relation is checked:

(14) $$i\partial_{t}\Psi +\mu \partial_{l}\partial^{l}\Psi =-\left[\partial_{t}\phi +\mu {\left(\partial_{l}\phi \right)}^{2}-\mu \frac{\partial_{l}\partial^{l}A}{A}\right]+\frac{i}{2A^{2}}\left[\partial_{t}A^{2}+2\mu \partial_{l}\left(A^{2}\partial^{l}\phi \right)\right]$$

Now, the “specific constraints” necessary for Ψ to be a solution of the non-stationary differential Equation (14) will be reducible to the differential equations:

(15) $$\begin{array}{c} \partial_{t}\phi +\mu \left(\partial_{l}\phi \partial^{l}\phi \right)=\mu \frac{\partial_{l}\partial^{l}A}{A}\\ \partial_{l}A^{2}+2\mu \left(A^{2}\partial_{l}\phi \right)=0 \end{array}$$

Moreover, with the notation:

(16) $$V_{D}^{i}=2\mu \partial^{i}\phi ,\;\rho =A^{2},\;V_{D}^{i}=\mu \partial^{i}\ln\rho$$

where $V_{D}^{i}$ is the differentiable component of the velocity field, $V_{F}^{i}$ is the non-differentiable component of the velocity field, and ρ is the density of states, the multifractal conservation law of the specific momentum is:

(17) $$\partial_{t}V_{D}^{i}+V_{D}^{l}\partial_{l}V_{D}^{i}=-\partial^{i}Q$$

and, respectively, the conservation law of the multifractal states density is:

(18) $$\partial_{t}\rho +\partial^{l}\left(\rho V_{D}^{l}\right)=0$$

with specific multifractal potential:

(19) $$Q=-\mu^{2}\frac{\partial_{l}\partial^{l}\sqrt{\rho }}{\sqrt{\rho }}=\frac{V_{F}^{l}V_{F}^{l}}{2}-\frac{\lambda }{2}\partial^{i}V_{F}^{i}$$

Through the induced specific multifractal force:

(20) $$f^{i}=-\partial^{i}Q=-\mu^{2}\partial^{i}\left(\frac{\partial_{l}\partial^{l}\sqrt{\rho }}{\sqrt{\rho }}\right)$$

becomes a measure of the fractal degree pertaining to the motion curves. In such a motion, the “specific constraints” (15) are also checked in detail, with a specific multifractal potential:

(21) $$Q\left(x,t\right)=\mu v\left(kx-v^{2}t^{2}\right)$$

suppressing, in the SRT sense, dynamics with a constant multifractal force.

The synergy of the two frameworks (Schrodinger and Madelung) for the LPP dynamics multifractal representation allows for the exploration over a select set of variables, as key parameters in the description of LPP at various scale resolutions. Therefore, at arbitrary differentiable-scale resolutions, the following definitions can be defined:

- differentiable velocity:
  (22) $$V_{D}=2\mu \frac{d\Phi }{dx}=2\mu^{2}k^{3}t$$
- differentiable current density:
  (23) $$J_{D}=\rho V_{D}=2\mu^{2}k^{3}t{\mathrm{Ai}}^{2}\left(kx-\mu^{2}k^{4}t^{2}\right)$$
- specific multifractal potential:
  (24) $$Q=\mu^{2}k^{2}\left(kx-\mu^{2}k^{4}t^{2}\right)$$
- global current density dependence on the specific multifractal potential:
  (25) JD=Ai2(Qμ2k2)×(μ2k3x−Qμ2k4)12,μ2k3x≥Q

While, at a nondifferentiable-scale resolution, the following set of variables are available:

- nondifferentiable velocity:
  (26) $$V_{F}=\mu \frac{d\ln\rho }{dx}=\frac{2\mu }{{\mathrm{Ai}}^{2}\left(kx-\mu^{2}k^{4}t^{2}\right)}\left[\frac{d\mathrm{Ai}\left(kx-\mu^{2}k^{4}t^{2}\right)}{dx}\right]$$
- nondifferentiable current density:
  (27) $$J_{F}=\rho V_{F}=2\mu \mathrm{Ai}\left(kx-\mu^{2}k^{4}t^{2}\right)\left[\frac{d\mathrm{Ai}\left(kx-\mu^{2}k^{4}t^{2}\right)}{dx}\right]$$
- plasma temperature, based on VF2=kBTm0:
  (28) $$T=\frac{4\mu^{2}m_{0}}{k_{B}}\frac{1}{{\mathrm{Ai}}^{2}\left(kx-\mu^{2}k^{4}t^{2}\right)}{\left[\frac{d\mathrm{Ai}\left(kx-\mu^{2}k^{4}t^{2}\right)}{dx}\right]}^{2},$$
  with $k_{B}$ as the Boltzmann constant and $m_{0}$ as the rest mass of the plasma structural unit.

### 2.2. LPP Dynamics in a Multifractal Framework

**Figure 6:**
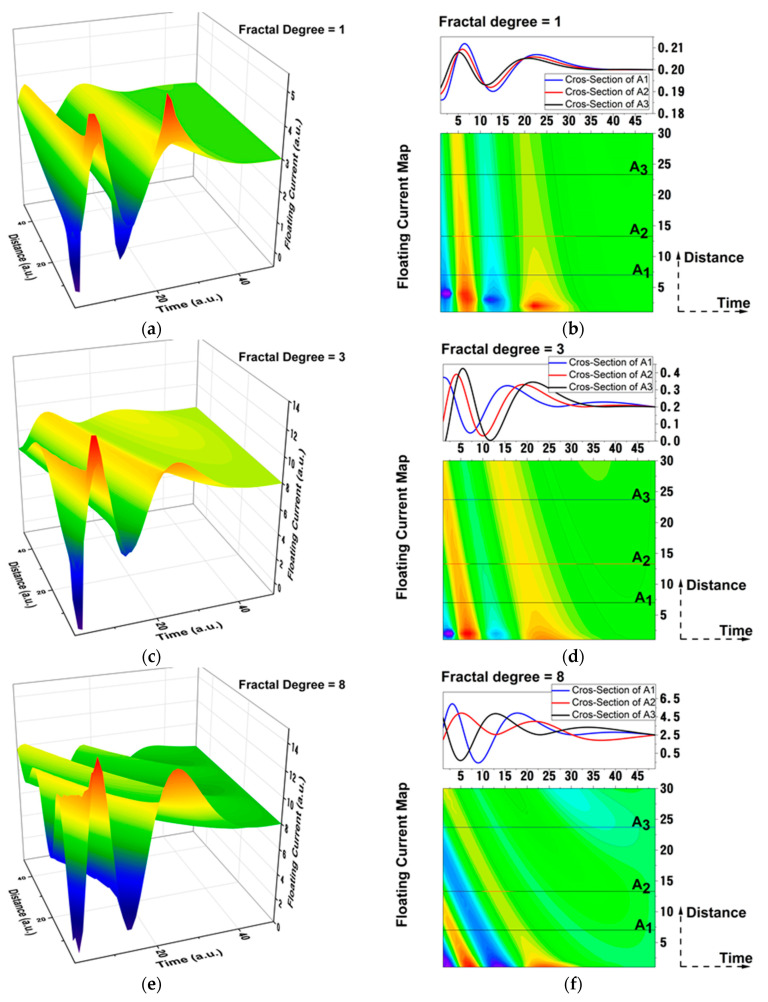

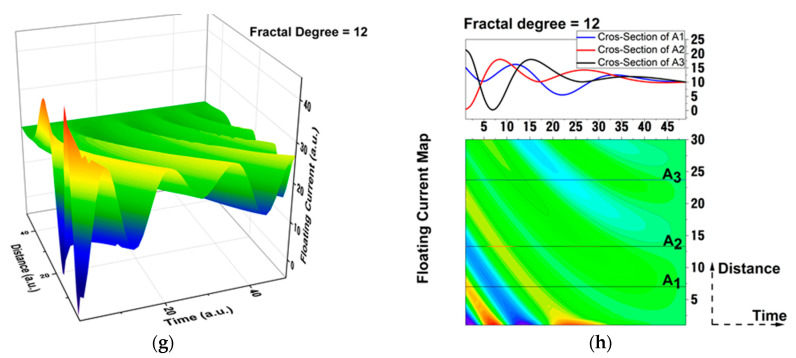
Space–time maps (1-(**a**); 3-(**c**); 8-(**e**); 12-(**g**)) of the floating current in 3D and contour plot representations (1-(**b**); 3-(**d**); 8-(**f**); 12-(**h**)) for various fractalization degrees (1, 3, 8, 12).

In Figure 6, we have represented the 3D and contour plot representation of the plasma floating current in a multifractal representation (23). Systematic simulations were performed for various fractal degrees. Each fractal degree encompasses a complex array of interactions from collisions, excitations, ionization, and even reactions with the background gas during expansion. The spatial and temporal evolution reveals the presence of two maxima. These two maxima can be correlated with a bunch of charges with different ionization degrees, as it was reported in [32,45], or attributed to a dual ablation mechanism contribution to the ionic cloud. The cross-sections performed on the contour plot representations show that there is a shift towards lower arrival times of the maxima as the distance is increased. This means that, during expansion for lower fractalization degrees, ions acceleration during expansion is achievable. Similar results were proposed by Bulgakova et al. in [10,46], where they reported on ion acceleration by the double layer present in the front of the plume. The increase in the fractalization degree is correlated with the increased number of interactions of the ablated particles and, thus, background gas pressure increase. The model shows that, with the increase of the background pressure, it is possible to create new groups of ions in the plasma with lower energies. This can be seen in A3 line for the fractalization degree of 3 and becomes more evident for simulated plasma with fractal degrees higher than 5. If the number of interactions is increased even more (the fractalization degree = 10 case), we observe the appearance of fast peaks at relatively large distances in the plasma. This means that the background gas is ionized by the plasma front, leading to the formation of a new ionic group in the plasma. This result is in line with the results from [26], where experimental proof of gas ionization during pulsed laser deposition was shown. 

**Figure 7:**
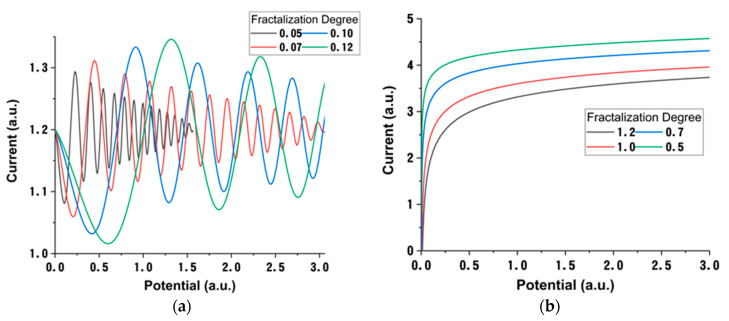
Current dependence with fractal potential in various domains of fractal degree <0.2 (**a**) and >0.5 (**b**).

Once established that the multifractal model can generate temporal traces of the charges in the plasma, we will change the focus towards characterizing the measurable properties of the plasma. Therefore, by using the approach from (Equation (25)), we have obtained a law that described the variation of the saturation charge as a function of the multifractal potential. The multifractal potential, in the framework of our model, acts like an internal constrain of the plasma limiting particular kinetics. We have represented, in Figure 7, the saturation current, as defined in the multifractal paradigm variation with the fractal potential. The results show that, for small fractal degrees, there is a modulated-type function describing the current−potential relationship. A low fractalization degree would correspond to a free expanding plasma with limited interactions between particles and strong deviation from the ideal plasma conditions. Thus, *ideal plasma conditions*, from a multifractal perspective, would be achieved in a high fractalized medium. The changes in the fractalization degree of the system are also related to the nature of the ablated target, as properties such as evaporation temperature, particle mass or radius, and ionization potential are important factors that contribute to the overall value of the fractalization degree, as defined in this work. As the medium becomes more fractalized, it reaches optimum conditions, so that the classical approach to LP method can be implemented. This can be seen in Figure 7b, where the modulated behavior is replaced by an exponential increase, followed by a saturation regime. The results are in good agreement with the data presented in Figure 1.

**Figure 8:**
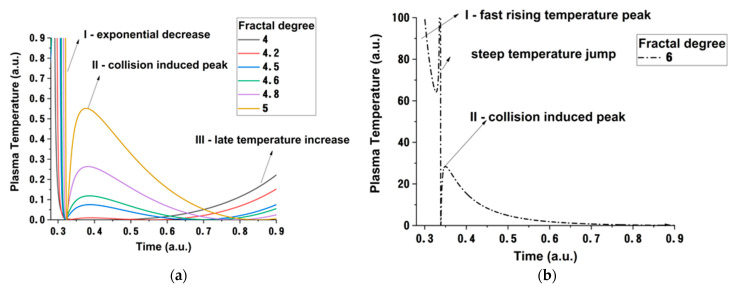
Temperature temporal evolution for various fractal degrees (**a**) and a particular evolution in conditions of high fractalization (**b**).

Finally, let us address the way a property such as plasma temperature is described in this multifractal framework. The understanding of plasma temperature, from the LP theory, offers a measurable information over the electron free*,* thermal movement in the plasma. The dynamics of electrons, especially in conditions of high background pressure, are dominated by collisions. In Figure 7, we showed that this is also a route to achieve thermodynamic equilibrium, which was presented in ref. [47], when by addition of Ar gas during LIBS measurement significant steps for establishing LTE were made. In the following, let us quantify the energy of the plasma through Equation (28), which represents the energy of the plasma induced by the internal forces acting on complex systems. In Figure 8, we have represented, by keeping a constant fractalization degree and having time as free parameter, the plasma temperature (in a multifractal paradigm understanding). For relatively lower fractalization degrees, we see that there are two contributions to the plasma temperature, one at short-time and a second one at later evolution times. This behavior is in line with the two-plasma structure scenario and can be related to the different natures of the main ablation mechanism. If the fractalization degree increases, we observed a third peak with a constantly increasing amplitude, and the one seen in the 0.7−0.9 regime was disappearing. This is a contribution to the plasma temperature induced through collision between the plasma and the background gas particles. Indeed, due to collisions, supplementary ionic groups can be induced in plasma, and the low energy ones can disappear they lose their kinetic energy fast and have become negligible to the overall plasma energy. This result is well in line with the experimental data presented in Figure 2 and previous reports of background gas effect on LPP dynamics [26]. In Figure 8b, we have represented the evolution of the temperature at a “extreme” value of the fractalization degree. We observe that, if the fractalization reached higher values, there are only two contributions: one at short-times, which is defined by an exponential decrease, and another at longer evolution times. These structures have a clear separation in the multifractal projection, and it appears as a decoupling in their interaction, as they do not influence each other anymore.

## 3. Materials and Methods

The 1-inch-high purity metallic targets (Co, Cu, Ag, and Bi) were irradiated with a YAG laser (λ = 266 nm, 5 ns) in identical conditions of repetition rate (10 Hz) and laser fluence (3.8 J/cm^2^). Continuous rotation of the targets was provided by a motorized stage, in order to prevent local heating and subsequent crater formation and provide fresh surface for each shot to mimic PLD conditions. A tungsten LP (diameter—0.2 mm and exposed length—2 mm) was placed at a distance of 37 mm, while a substrate was placed 50 mm from the target to keep the PLD geometry. Plasma investigations were performed at various Ar pressures, ranging from 5 × 10^−5^ Pa residual pressure to 5 × 10^−2^, 5 × 10^−1^, 2, 5, and 10 Pa Ar. A cleaning procedure, consisting of continuous irradiation of the target, with 1200 pulses, was implemented before each experiment. The LP was shielded from the incoming transient plasma during the cleaning procedure. A measurements procedure, following the step reported in [20], was implemented, in order to collect the ionic or electronic temporal traces by applying a wide range of biases (±20 V) and recording the voltage signal across a load resistor with a Tektronix DPO 4140 oscilloscope. All performed investigations were time-synchronized by a fast silicon photodiode (Thorlabs FDS100), and the initial measuring moment was the moment when the laser was fired.

## 4. Conclusions

The Langmuir probe method was evaluated for a selected range of plasmas, expanding in various Ar pressures. Sub-microsecond modulation was observed in the reconstructed *IV* characteristic, which was attributed to non-equilibrium dynamics of the ejected charges. This feature was present for all investigated plasmas and is defining a non-equilibrium expansion regime of the plasma as it vanishes, both with the expansion of time and Ar gas addition. Time resolved investigations revealed the presence of a perturbative regime, which was recorded for Ar gas pressures higher than 2 Pa, when the probe entered a region where ionic bursts appeared in the electron saturation region. Frequencies in the 20–60 kHz regimes were recorded, associated with ion plasma oscillations. The oscillations frequency was well-correlated with the atomic mass of the ablated metallic target. This perturbation is identified as a plasma fireball. Based on the behavior of the perturbation, the nature of the fireball was found to be a mixture of Ar and metal ions from the plasma. Both short- and long-time non-linear behavior were correlated via probe bias and pressure effects on the strange attractors defining the fireball-like behavior were investigated. A non-linear dynamic’s analysis was performed and the strange attractors characterizing each fireball were reconstructed. The attractors were defined by two branches, which become interconnected as the Ar gas pressure increased. The Ar atmosphere plays the role of a coherence medium, correlating short- and long-time nonlinear behavior. A non-linear multifractal model was developed here, in order to explore these new regimes of the LP measurements. Both wave-like behavior in time and potential space were simulated via multifractal dynamics. A good correlation was observed between the simulated data and experimental findings. 

## Data Availability

Data sharing is not applicable for this paper.
